# The molecular basis of chemoradiosensitivity in rectal cancer:implications for personalized therapies

**DOI:** 10.1007/s00423-012-0929-5

**Published:** 2012-03-02

**Authors:** Marian Grade, Hendrik A. Wolff, Jochen Gaedcke, B. Michael Ghadimi

**Affiliations:** 1 Department of General and Visceral Surgery, University Medical Center Göttingen, Robert-Koch Str. 40, 37075 Göttingen, Germany; 2 Department of Radiotherapy and Radiooncology, University Medical Center Göttingen, Robert-Koch Str. 40, 37075 Göttingen, Germany

**Keywords:** Rectal cancer, Preoperative chemoradiotherapy, Chemoradiosensitivity and chemoradioresistance, Response prediction, Molecular biomarker

## Abstract

**Introduction:**

Preoperative chemoradiotherapy represents the standard treatment for patients with locally advanced rectal cancer. Unfortunately, the response of individual tumors to multimodal treatment is not uniform and ranges from complete response to complete resistance. This poses a particular problem for patients with a priori resistant tumors because they may be exposed to irradiation and chemotherapy, treatment regimens that are both expensive and at times toxic, without benefit. Accordingly, there is a strong need to establish molecular biomarkers that predict the response of an individual patient’s tumor to multimodal treatment and that indicate treatment-associated toxicities prior to therapy. Such biomarkers may guide clinicians in choosing the best possible treatment for each individual patient. In addition, these biomarkers could be used to identify novel molecular targets and thereby assist in implementing novel strategies to sensitize a priori resistant tumors to multimodal treatment regimens.

**Objective:**

The aim of this review is to summarize recent findings about the molecular basis of treatment resistance and treatment toxicity in patients with rectal cancer. Whole-genome, as well as single-biomarker or multibiomarker, analyses and their potential implications will be highlighted. At the end, we will outline a future vision of rectal cancer treatment in the era of personalized medicine.

## Introduction

For rectal cancers, both surgical and nonsurgical treatment concepts have been improved considerably over the last decades. In this respect, the surgical concept of total mesorectal excision (TME) [1] and the implementation of preoperative treatment regimens can be considered as cornerstones of modern and optimized treatment for locally advanced rectal cancer. Randomized multicenter trials independently demonstrated that preoperative application of radiotherapy, either as short-term radiotherapy or as long-term irradiation accompanied by infusional 5-fluorouracil (5-FU), significantly decreases the rate of local recurrence [2, 3]. Consequently, preoperative (chemo)radiotherapy now represents the standard treatment for patients with locally advanced rectal cancer in Europe and the USA [4].

With respect to long-term preoperative chemoradiotherapy, it was soon realized that the histopathological response to chemoradiotherapy varied tremendously from one patient to another, ranging from complete regression to complete resistance, and that, for many patients, the extent of regression was correlated to clinical outcome [5]. In order to increase the response rates to preoperative chemoradiotherapy, agents such as oxaliplatin and antibody-based regimens have been incorporated into multimodal treatment concepts, and these strategies are currently under extensive evaluation [4]. However, at the same time, the inclusion of more toxic agents increased the acute toxicity and long-term side effects of these treatments [4]. In some cases, acute organ toxicity necessitates a dose reduction or even termination of therapy. Furthermore, any higher grade of acute toxicity impairs the quality of life of the individual patient.

While the variability of both treatment-associated tumor regression and toxicity from one patient to another remains a major clinical problem, it obviously draws attention to the important possibility to individualize rectal cancer treatment. In this respect, some patients may require an intensified regimen to increase tumor response, whereas standard 5-FU-based chemoradiotherapy may be sufficient for others. However, advancing accurate stratification is strongly dependent on the identification of reliable clinical parameters and molecular biomarkers that allow pretherapeutic stratification with high certainty. The aim of this review is to briefly summarize the general principles of chemoradiosensitivity and to highlight recent findings about the molecular basis of treatment response and toxicity in patients with rectal cancer. At the end, we will discuss potential implications of these findings for an individualized treatment of rectal cancer patients.

## General principles of chemoradiosensitivity

The predominant local effects of chemoradiotherapy, which is designed to achieve tumor cell damage, are primarily elicited by irradiation, whereas concomitant chemotherapy may serve as a radiosensitizer, most often without or with only small direct effects on tumor cell killing. The effects of chemoradiotherapy are largely the result of DNA damage, which either occurs directly through ionization within the DNA molecule or indirectly from the action of chemical radicals, which are also formed during irradiation [6, 7]. Through these mechanisms, several alterations, like base damage, DNA-protein cross-links, and single-strand or double-strand breaks, are generated and contribute to the antitumor effects and side effects [8].

However, as already discussed above, response to chemoradiotherapy differs widely. In general, four major explanations for these different sensitivities of solid tumors have been invoked, which could be causative alone or in combination:

1. Tumors with poor response to chemoradiotherapy often show a high content of hypoxic cells and/or fail to reoxygenate during fractionated multimodal treatments. For this reason, the necessary DNA damage caused by chemical radicals formed as a result of local ionization is not sufficient to kill all tumor cells. In general, this fact prevails with increasing tumor sizes including larger hypoxic and necrotic areas with lower oxygen pressure [9, 10].
2. It is well known that cells located in proliferating cell cycle segments are more sensitive to chemoradiotherapy compared to cells resting or degenerating. Therefore, the extent of tumor regression during treatment may be explained in part by the proportion of proliferating cells. A higher growth fraction in solid tumors might lead to a larger turnover rate with a higher proportion of cell loss in comparison to normal tissues [11].
3. Varying levels of repair capacity of DNA damage caused by chemoradiotherapy have been specified in almost all tumor cell lines and found to correlate with the clinical radiocurability. In other words, less curable tumors often show a strong potential for DNA damage recovery. A confirmation of this fact in larger clinical trials, which could justify its use as predictive marker for tumor response and outcome, has not been achieved so far. This might in part be due to the fact that any given tumor always harbors a mixed cell population, with mitotic or resting cells and stem cells with differing sensitivity to antineoplastic agents and irradiation-induced damage [12].
4. There seems to be an inherent chemoradiosensitivity, which is associated with the individual genetic sensitivity of every single patient. Assuming that all malignant cells arise from formerly normal tissue cells, it should thus be possible to determine the individual response to multimodal therapies via the patient’s individual genetic profile [13]. This fact is underlined by recent literature clearly showing a correlation between acute organ toxicity, which is a measure of inherent chemoradiosensitivity, and outcome for different tumor entities. For example, overall survival in patients with inoperable head and neck cancer was significantly associated with treatment-related high-grade acute organ toxicity [14].

## Molecular basis of chemoradiosensitivity in rectal cancer

Despite the clinical importance of preoperative chemoradiotherapy in multimodal treatment concepts for patients with rectal cancer, our understanding of both the genetic basis of chemoradiosensitivity and the molecular events leading to chemoradioresistance remains relatively sparse. Relevant investigations that have been performed to identify molecular biomarkers differentiating responsive and resistant tumors will be discussed below. From a systematic point of view, high-throughput analyses (aim: whole-genome analysis) can be distinguished from low-throughput analyses (aim: single-biomarker or multibiomarker analysis).

Since complex phenotypes, such as tumor responsiveness to chemoradiotherapy, likely do not depend on the alteration or deregulated expression of single genes, high-throughput technologies have emerged as a central tool in deciphering the molecular basis of this clinically important phenotype because they offer the possibility to identify genomic differences between two groups of patients. However, due to the high number of observed genomic features, it represents a nontrivial task to determine which of these features are actually relevant, and this kind of analysis generally requires a high number of patients. Therefore, in situations where there is already prior biological knowledge pointing to a certain biomarker of interest or when preliminary evidence suggests the involvement of certain pathways, it can be advantageous to focus on a single or few selected biomarkers. Such studies, with a limited number of biomarkers of interest, require lower case numbers in order to reach statistical significance since correction for multiple testing, which is mandatory for high-throughput analyses, can be omitted [15].

### Whole-genome analyses

#### Gene expression profiling

Expression microarrays are commonly used to comprehensively interrogate complex genetic pathways and networks [16, 17]. Consequently, several investigators have used gene expression profiling to analyze the genetics of (colo)rectal cancer response to chemoradiotherapy (recently reviewed by Kuremsky et al. [18], Nannini et al. [19], and Brettingham-Moore et al. [20]). From a systematic point of view, in vivo studies (profiling of primary rectal cancers) need to be examined separately from in vitro studies (profiling of cancer cell lines).

##### In vivo studies

In a first published report, pretherapeutic biopsies from 30 patients with locally advanced rectal carcinomas were profiled using a cDNA microarray [21]. All patients participated in the CAO/ARO/AIO-94 trial of the German Rectal Cancer Study Group (GRCSG) [3]. Tumor response was defined as T-level downsizing, and a set of 54 genes was found to be differentially expressed between responsive and resistant tumors. Using a leave-one-out cross-validation strategy, response was correctly predicted in 83% of patients. A follow-up study indicated that these genes could also assist in predicting local recurrence and disease-free survival [22]. These data are currently being validated in a much larger set of patients (*n* > 200) who participated in the CAO/ARO/AIO-04 trial of the GRCSG, treated within different institutions. Preliminary analyses of this cohort confirm the effectiveness of expression profiling for predicting outcome (Gaedcke and Ghadimi, unpublished data).

Shortly thereafter, a Japanese group published a gene expression analysis of 52 rectal cancer patients treated with preoperative radiotherapy [23]. They reported that 33 genes were differentially expressed between responders and nonresponders (based on histopathological regression grading of surgically resected specimens), with a class prediction accuracy of 88.6%.

Kim and colleagues described the identification of 261 genes that were differentially expressed between 20 partial responders to preoperative chemoradiotherapy, based on tumor regression grading, and 11 complete responders (defined as the training set). This set was validated in a test set of 15 patients. The authors reported class prediction accuracies of 84% (training set) and 87% (test set), respectively [24].

Similarly, Rimkus and colleagues profiled pretherapeutic biopsies from 43 patients with locally advanced rectal cancers. Using histopathologic response as endpoint for comparison with gene expression, they identified a 42-gene signature [25]. In the most recent study, Brettingham-Moore and colleagues analyzed pretherapeutic biopsies from 51 locally advanced rectal cancers and generated gene expression classifiers based on tumor regression grade, metabolic response, and UICC downstaging [26]. The sensitivity and specificity of these classifiers to predict outcome after preoperative chemoradiotherapy centered around 82% and 89%, respectively. Interestingly, the authors also tested the effectiveness of previously published gene expression signatures to predict outcome in their data set, but the results were rather unfruitful.

It is important to note that there was only a very limited overlap of genes from within these signatures. This could be due to several reasons: *First*, there are many different ways of defining “response” and “resistance.” *Second*, case numbers were different. *Third*, there are differences in the number and identity of spotted genes on the respective microarrays. *Fourth*, there is the problem of high dimensionality of the data. In most settings, the number of patients is naturally limited (i.e., in the order of tens or hundreds), while the number of measured features, i.e., gene transcripts, is usually very high (in the order of thousands or tens of thousands). It is, therefore, very likely that several gene expression signatures exist that are able to accurately predict the clinical outcome. This may be particularly true since genes are often highly interrelated, and homologs or isotypes may serve similar functions in different pathways. In the process of selecting a gene expression signature, it is, therefore, sometimes a matter of random choice which genes will end up in the classification signature and which genes will not. In the past, this has lead to enormous confusion and debate in the field [27–29]. Some more recent methods actually try to avoid this problem by guiding the gene selection process using prior knowledge, based, for example, on pathway and functional network databases [30, 31].

Despite these limitations, gene expression analyses hold considerable promise to unveil the underlying complex genetics of chemoradioresistance and to play a future role in stratifying rectal cancer patients. In this respect, breast cancer constitutes a prominent biological precedent to demonstrate the feasibility of using expression signatures in clinical decision-making (proof-of-principle). For this disease entity, a prognostic signature, consisting of 70 genes, has been established [32, 33] and subsequently extensively validated [34, 35], resulting in the initiation of a multicenter trial confirming the clinical effectiveness of this gene set (“Microarray in Node-Negative Disease May Avoid Chemotherapy” [MINDACT] trial).

##### In vitro studies

Cancer cell lines are widely used as model systems for target screening, drug discovery, and functional analyses because many features of primary tumors are recapitulated in derived cell lines [36–39]. Eschrich and colleagues were the first to report a gene expression-based model for in vitro sensitivity of colorectal cancer cell lines to irradiation, although their analysis only included seven colorectal cancer cell lines [40, 41]. The remaining 41 cell lines were derived from different entities, including breast, ovarian, renal, prostate, and non-small cell lung cancers, leukemia, melanoma, and tumors of the central nervous system. Building on their own earlier work [42], the authors integrated the respective surviving fractions of these 48 cell lines after irradiation at 2 Gy by pretreatment gene expression profiles, *KRAS* and *TP53* mutation status, and tissue of origin and extracted a network of 10 signature genes. This expression model of intrinsic radiosensitivity, which included prominent target genes such as *JUN*, *STAT1*, and *CDK1*, was subsequently validated in three cohorts of patients with rectal, esophageal, and head and neck cancer [41].

In a similar study, Spitzner and colleagues reported the identification of a gene expression signature for sensitivity of colorectal cancer cell lines to chemoradiotherapy [43]. A panel of 12 cell lines was exposed to doses of both 5-FU and radiation that were similar to the ones used in the clinic, i.e., 3 μM of 5-FU and 2 Gy of radiation, and the respective surviving fractions were correlated with pretreatment gene expression profiles. Analysis of this chemoradiosensitivity signature revealed many genes involved in mitogen-activated protein kinase (MAPK), insulin, and Wnt signaling, cell cycle genes, and novel potential target genes such as *STAT3* or *ERBB2* [43].

As already pointed out for primary tumors (see the “In vivo studies” section), there is only a very limited overlap between the respective in vitro sensitivity signatures. In addition to the reasons discussed above, there are other potential explanations. *First*, Spitzner and colleagues correlated gene expression and sensitivity to both chemotherapy and irradiation, while Eschrich and colleagues established a signature of radiosensitivity. *Second*, Eschrich and colleagues included cell lines that were mismatch repair (MMR) proficient as well as cell lines that were MMR deficient, although these pathways are genetically different [44, 45]. *Third*, Eschrich and colleagues analyzed a panel of cell lines from various tumor entities.

#### Chromosomal aberrations

Chromosomal aneuploidy is a defining feature of colorectal carcinomas [46, 47]. This is reflected by tumor- and stage-specific genomic copy number aberrations [48], which are virtually identical in colon and rectal cancers [49, 50]. Accordingly, it may be speculated that differences in treatment responses can be correlated with differences on the DNA level.

In one of the first studies to address this question, pretherapeutic biopsies from 42 patients with locally advanced rectal cancers were analyzed using metaphase comparative genomic hybridization (CGH). Based on downsizing of the T-category, chromosomal gains of 7q32–q36 and 7q11–q31 as well as amplifications of 20q11–q13 were associated with responsiveness to preoperative chemoradiotherapy [51]. However, the authors reported a high probability that these genomic copy number changes were detected by chance, therefore requiring independent validation in a larger patient population and with a higher resolution. In a more recent study, Chen and colleagues used oligonucleotide array-based CGH to screen for chromosomal copy number alterations correlated with pathologic complete response (pCR). Analyzing DNA from 95 rectal cancers, the authors observed that chromosomal loss of 15q11.1–q26.3 was associated with non-pCR, while loss of 12p13.31 was associated with pCR [52].

### Single-biomarker and multibiomarker analyses

#### DNA mutations in the RAS–MAPK pathway

The *v-Ki-ras2 Kirsten rat sarcoma viral oncogene homolog* (*KRAS*), a member of a large family of GTP-binding proteins involved in signal transduction [53, 54], plays an important role in colorectal carcinogenesis because a high percentage of colorectal carcinomas are characterized by activating mutations of this oncogene [45]. These mutations result in the constitutive activation of the MAPK pathway. Because preliminary evidence indicated an ability of the *RAS* oncogene to enhance radioresistance in vitro [55–58], several studies investigated the potential relevance of the RAS–MAPK pathway for the response of primary rectal cancers to irradiation.

Luna-Perez and colleagues correlated the response of 37 rectal cancers to preoperative chemoradiotherapy with *KRAS* mutation status [59]. Analyzing codons 12, 13, and 61, they could show that tumors with wild-type *KRAS* were more likely to be responsive than tumors with mutant *KRAS*. It should be noted that the authors used irradiated tumor tissue for their analysis, although recent evidence suggests that preoperative multimodal treatment does not alter *KRAS* mutation status [60]. In contrast, Zauber and colleagues screened pretherapeutic biopsies from 53 patients with stage I–III rectal cancers and detected *KRAS* mutations in 18 patients (34%). The presence of a *KRAS* mutation, however, was not indicative of tumor regression after preoperative chemoradiotherapy [61].

Shortly thereafter, Gaedcke and colleagues observed a *KRAS* mutation frequency of 48% (*n* = 45) in pretherapeutic biopsies of 94 patients with locally advanced rectal cancers [62]. In contrast to Zauber and colleagues, these authors specifically reported the affected codons: Twenty-nine mutations (64%) were located in codon 12, 10 mutations (22%) in codon 13, and 3 mutations (7%) each in codons 61 and 146. The presence of none of these mutations was correlated with response to preoperative chemoradiotherapy. However, Gaedcke et al. detected differential sensitivities when the mutations were grouped based on the respective amino acid exchange; G12V mutations appeared to be associated with higher rates of tumor regression than G13D mutations (*p* = 0.012). Most recently, Garcia-Aguilar and colleagues published their analysis of pretherapeutic biopsies from 132 patients with locally advanced rectal cancers and reported that *KRAS* mutations were more likely in tumors from patients without pCR, i.e., resistant tumors [63].

These conflicting data indicate that it may not be sufficient to solely determine the mutation status of *KRAS*, but rather to group patients according to the respective nucleotide-specific amino acid exchange. This interpretation is supported by previous investigations suggesting that the level of aggressiveness depends on the mutation form [64, 65] and that these specific mutation forms may activate distinct downstream targets and different oncogenic pathways [66]. Furthermore, De Roock and colleagues recently reported that chemotherapy-refractory metastatic colorectal cancers harboring a G13D *KRAS* mutation were more sensitive to treatment with the epidermal growth factor receptor (EGFR) inhibitor cetuximab compared to tumors with other *KRAS* mutations [67]. This was confirmed in vitro, and G12V-mutated cancer cells were resistant to cetuximab, whereas G13D-mutated and *KRAS* wild-type cancer cells were sensitive. Whether the *KRAS* mutation status also influences the response of rectal cancers to multimodal treatment concepts that include EGFR inhibitors remains elusive, particularly as the clinical relevance of these combinations remains to be determined [68, 69].

#### Single-nucleotide polymorphisms

Single-nucleotide polymorphisms (SNPs) are sites in the genome sequence where individuals differ by a single base [70]. The total number of these sites in the human genome is estimated to be roughly 10 million, and these SNPs are distributed at an overall frequency of 1 in every 300 to 1,000 base pairs [71]. Importantly, it has been demonstrated that specific haplotypes and genetic polymorphisms are associated with clinical phenotypes. For instance, the presence of a G allele within the SNP rs6983267, located on chromosome 8q24, confers an increased risk for the development of colorectal cancer [72–74]. Due to the growing body of evidence suggesting that genetic variation between individuals can account for differences in drug response [75, 76], it has been speculated that genetic polymorphisms in genes encoding drug- or radiation-related responses may influence the individual’s response to chemoradiotherapy [77].

Most prominently, thymidylate synthase (TS) has been analyzed in this respect, but the results are conflicting. Villafranca and colleagues were the first to correlate polymorphisms in the *TS* promoter and tumor response to preoperative chemoradiotherapy. Analyzing tumor DNA from pretreatment biopsies of 65 patients, the authors observed that the *TS* genotype was predictive for tumor downstaging following preoperative chemoradiotherapy [78].

Terrazzino and colleagues, as well as the other investigators mentioned within this section, analyzed germline (blood) DNA from rectal cancer patients. However, there was no correlation with histopathological tumor regression [79]. This result has recently been confirmed by Conradi and colleagues who also failed to demonstrate any association between *TS* genotype and relevant clinical parameters such as local response, tumor regression grading, or disease-free and overall survival [80].

In contrast, Spindler and colleagues demonstrated that the *TS* genotype had a significant impact on the rate of complete pathological response following preoperative chemoradiotherapy [81]. Similar results were subsequently published by Stoehlmacher and colleagues who also reported a correlation between *TS* genotype and histopathological tumor regression [82]. Data from Hur and colleagues confirm the interpretation that SNPs within the *TS* enhancer region affect the response of rectal cancers to preoperative chemoradiotherapy [83]. Very recently, Tan and colleagues reported the prospective use of *TS* genotyping to direct preoperative chemoradiotherapy in a single-institution phase II study [84].

As a second example, recent data indicated that germline polymorphisms in the *TGFB1* gene are associated with quality of life-impairing acute organ toxicity in patients with locally advanced rectal cancer. Analyzing DNA from two independent cohorts of patients participating in the CAO/ARO/AIO-94 and -04 trials (*n* = 88 and *n* = 75), Schirmer and colleagues demonstrated that all patients carrying the *TGFB1* Pro25 variant developed high-grade acute organ toxicity during preoperative 5-FU-based chemoradiotherapy [85]. The positive predictive value for acute toxicity in the presence of this SNP is 100%, which highlights the potential clinical importance of this observation.

#### Immunohistochemistry

A plethora of studies has been published which focused on a single immunohistochemical marker or a combination of a few. A comprehensive summary of these studies would extend beyond the scope of this review, and we refer the reader to recent comprehensive reviews [18, 86, 87]. Very briefly, primary focus was the analysis of proteins involved in DNA damage repair, proliferation, angiogenesis, and apoptosis, including Ki-67, cyclin E, p21, p53, survivin, Bcl-2, BAX, EGFR, VEGF, PCNA, XIAP, PTGS2 (COX-2), HIF-1α, TS, and PROM1 (CD133). However, for most marker studies, the results are conflicting and still remain inconclusive.

## Novel molecular targets for chemoradiosensitization

As discussed above, there is a clinical need to establish molecular biomarkers that differentiate responsive and resistant tumors because such biomarkers could be used pretherapeutically to predict the response of an individual patient’s tumor to multimodal treatment (*diagnostic approach*). In addition, genes that are differentially expressed between resistant and responsive tumors could be used to identify novel therapeutic targets and thereby assist in implementing novel therapeutic strategies (*therapeutic approach*). For instance, genes that are overexpressed in resistant tumors could be repressed via RNA interference (RNAi)-based approaches [88, 89] or using chemical/small molecule inhibitors, potentially leading to sensitization to chemoradiotherapy. In this context, both survivin and T cell-specific factor 4 (TCF4) represent two prominent and promising examples.

### Survivin

The *baculoviral inhibitor of apoptosis repeat-containing 5* (*BIRC5*), more commonly referred to as *survivin*, encodes for the smallest and structurally unique member of the inhibitors of apoptosis family of proteins [90]. Survivin is overexpressed in a variety of human tumors, and it plays a prominent role in regulating apoptosis, during cell division, and during adaptation to stress.

Following up on the observation that the expression of survivin was inversely correlated with spontaneous and radiation-induced apoptosis [91], Rödel and colleagues used siRNA-mediated gene silencing to demonstrate that inhibition of *survivin* sensitizes colorectal cancer cells to radiation therapy, accompanied by increased levels of G2/M phase arrest and increased levels of DNA double-strand breaks after irradiation [92]. Very recently, the same authors could show that survivin rapidly accumulates in the nucleus following irradiation where it subsequently interacts with members of the DNA double-strand break repair machinery in order to regulate the activity of DNA-dependent protein kinase [93]. Because survivin inhibitors are currently being investigated in clinical trials, future studies will ultimately demonstrate whether its inhibition represents an effective strategy for (chemo)radiosensitization [94]. In this respect, the potential relevance of survivin for monitoring response to preoperative chemoradiotherapy has recently been confirmed by Sprenger and colleagues who could show that high survivin expression in pretreatment biopsies correlated with advanced posttherapeutical tumor and UICC stage and decreased disease-free survival [95].

### TCF4

As discussed above, Ghadimi and colleagues reported the identification of a 54-gene signature that differentiated resistant and responsive rectal cancers from patients who had been treated with preoperative chemoradiotherapy [21]. Interestingly, within this signature, the transcription factor *TCF4* was found to be significantly overexpressed in resistant tumors. TCF4, also known as TCF7L2, represents a key downstream effector that mediates canonical Wnt signaling, a pathway that plays a central role in colorectal tumorigenesis and tumor progression [96, 97].

**Fig. 1:**
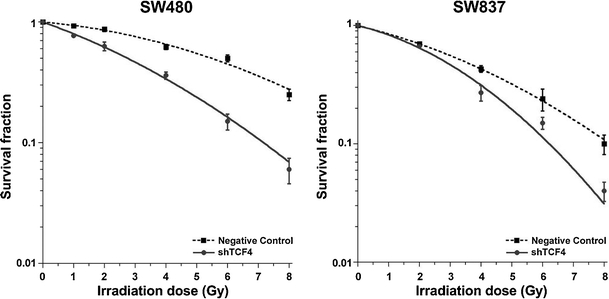
RNAi-mediated silencing of *TCF4* results in radiosensitization. *TCF4* was silenced in SW837 and SW480 cells using shRNA constructs, and stable single-cell clones were subsequently established. A standard colony-forming assay demonstrated that silencing of *TCF4* significantly increased the sensitivity of SW480 and SW837 cells to clinically relevant doses of X-rays

In order to explore the functional relevance of this overexpression for mediating treatment resistance, Kendziorra and colleagues recently silenced *TCF4* in resistant colorectal cancer cell lines and could show that RNAi-mediated inhibition of *TCF4* caused a significant radiosensitization of colorectal cancer cells with high TCF reporter activity (Fig. 1). Follow-up experiments revealed that the effect of radiosensitization was associated with a G2/M phase arrest, an impaired ability to adequately halt cell cycle progression after irradiation, and a compromised DNA double-strand break repair [98]. These data indicate a novel role of the Wnt transcription factor *TCF4* in mediating radioresistance and, if further validated, suggest that *TCF4* is a promising therapeutic target.

## Conclusions and future perspective

**Fig. 2:**
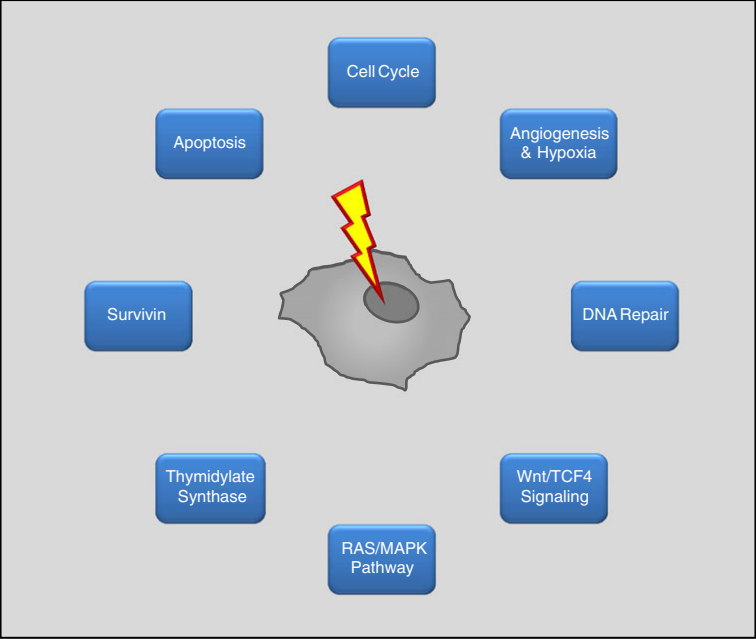
Potential pathways and proteins regulating and mediating resistance of rectal cancer cells to chemoradiotherapy

The genetic diversity of rectal cancer is associated with varying responses to chemoradiotherapy, and varying toxicity rates. This offers a wide range of options to pretherapeutically assess both response and toxicity for the individual patient. Consequently, a plethora of potential biomarkers has already been evaluated using whole-genome and single-marker or multimarker analyses, some of which have great potential to stratify rectal cancer patients for multimodal treatment regimens and to implement targeted therapeutics (Fig. 2).

However, there are several drawbacks of these findings that still impede transition to routine clinical practice: *First*, conflicting results were obtained by different investigators. *Second*, virtually all biomarkers described to date have been identified in retrospective studies and lack independent validation in a prospective setting using standardized analytical procedures. This represents the most challenging hurdle to the implementation of these biomarkers, once further validated, into a clinical setting.

**Fig. 3:**
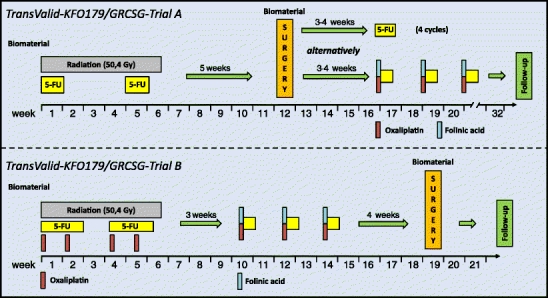
Outline of the TransValid-KFO179/GRCSG-Trials (TransValid A, TransValid B). *TransValid A* (*validation study*): 200 patients will be treated with 5-FU-based (1,000 mg/m^2^, 120 h continuous i.v. on days 1–5 and 29–33) chemoradiotherapy (radiation, 28 × 1.8 Gy) followed by radical surgery. Adjuvant therapy consists of either four cycles of 5-FU (500 mg/m^2^, bolus i.v. on days 1–5, repeat on day 29) or, in selected cases based on the clinicians’ discretion, six applications of a shortened FOLFOX regimen (folinic acid 400 mg/m^2^, 2 h continuous i.v.; oxaliplatin 100 mg/m^2^, 2 h continuous i.v.; 5-FU 2,400 mg/m^2^, 46 h continuous i.v.; on days 1, 15, 30, 45, 60, and 75). *TransValid B* (*feasibility study*, *phase I/II*): 50 patients will be treated with chemoradiotherapy (radiation, 28 × 1.8 Gy; 5-FU 250 mg/m^2^, continuous i.v. on days 1–14 and 22–35; oxaliplatin, 50 mg/m^2^, 2 h continuous i.v. on days 1, 8, 22, and 29), followed by three applications of a shortened FOLFOX regimen on days 1, 15, and 30 and radical surgery

In this respect, the TransValid-KFO179/GRCSG-Trials (TransValid A, TransValid B) are the first biomarker-driven clinical trials for patients with rectal cancer (Fig. 3). Funded by the Deutsche Forschungsgemeinschaft and initiated and promoted by the institutions of the GRCSG as well as the Clinical Research Unit 179 (Klinische Forschergruppe, KFO179), the aim of these multicenter studies is to prospectively implement the validation of previously identified molecular and clinical biomarkers into a highly standardized clinical setting. To achieve this goal, patients with locally advanced (cUICC II/III) cancers of the lower two thirds of the rectum as well as patients with resectable synchronous liver metastases (UICC IV) can be enrolled into one of these trials based on the responsible clinicians’ discretion. In TransValid A (“validation study”), patients are treated with standard 5-FU-based chemoradiotherapy followed by standardized TME surgery according to the German S3 Guideline (Fig. 3). Adjuvant chemotherapy consists of either 5-FU monotherapy or, in selected cases based on the clinicians’ assessment, a shortened FOLFOX regimen. The aim of TransValid B (“feasibility study,” phase I/II) is to establish the feasibility of an intensified preoperative chemoradiotherapy regimen (radiation, 5-FU, and oxaliplatin) combined with a shortened FOLFOX regimen prior to standardized TME surgery (Fig. 3). One rationale for this approach is the fact that today many patients do not receive, or receive limited doses of, adjuvant chemotherapy. Ascertainment of biomaterial at various time points is a major inclusion criterion for the TransValid trials (Fig. 3). This biomaterial will be processed according to previously established strict standard operating procedures, which includes performing these experiments on a week-by-week basis (prospective data generation).

**Fig. 4:**
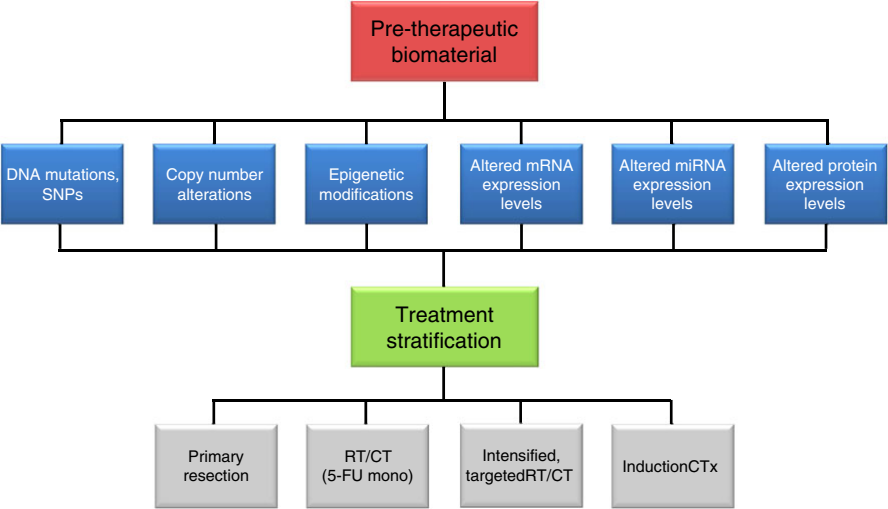
Future vision for the treatment of patients with locally advanced rectal cancers. Pretherapeutic patient material (tumor and normal tissue) will be subjected to multilayer genomic analyses. Based on the results of these analyses, patients will be stratified into different (preoperative) treatment concepts (personalized medicine)

From a personal perspective, we strongly believe that molecular biomarkers will be implemented into clinical decision-making in the near future. In a potential scenario, pretherapeutic patient material from both tumor and normal tissue will be ascertained at the initial diagnosis and subjected to multilayer genomic analyses (Fig. 4). Based on the results of these analyses (aim: prediction of both response and toxicity), the individual patient will be stratified into different alternative treatment concepts (personalized medicine). In this setting, patients with a biomarker profile indicating “responder to standard treatment” are subjected to a low-toxicity preoperative regimen. In contrast, for patients with a biomarker profile indicating “nonresponder to standard treatment,” a more aggressive approach is needed. For instance, an intensified regimen could be pursued, including the application of more effective systemic agents such as oxaliplatin. An induction combination chemotherapy (preoperative chemoradiotherapy followed by chemotherapy with sufficient dose and intensity prior to surgery) would be another interesting option because many patients do not receive adjuvant chemotherapy after preoperative chemoradiotherapy and surgical resection (either due to surgical complications, patients’ refusal, or investigators’ discretion). For patients predicted to be “nonresponder to standard treatment” and to develop high acute organ toxicity, primary surgery may be an option. With respect to novel therapeutic target genes, there are examples of molecular targets such as survivin and TCF4 that have the potential to be incorporated into treatment concepts, although this requires extensive validation and testing.

In any case, rectal cancer represents a prominent example on how to individualize multimodal treatment regimens. Once demonstrated that predictive biomarkers can be examined in a week-by-week setting with high quality and reproducibility and in a cost-effective manner, and once these biomarkers have been prospectively validated utilizing sufficient patient numbers, a personalized medicine is within reach. This holds considerable promise to improve the outcome of patients with this disease.
